# A New Real-Time Simple Method to Measure the Endogenous Nitrate Reductase Activity (Nar) in *Paracoccus denitrificans* and Other Denitrifying Bacteria

**DOI:** 10.3390/ijms25189770

**Published:** 2024-09-10

**Authors:** José J. García-Trejo, Sharon Rojas-Alcantar, Monserrat Alonso-Vargas, Raquel Ortega, Alejandro Benítez-Guzmán, Leticia Ramírez-Silva, Natalia Pavón, Claudia Peña-Segura, Ofelia Méndez-Romero, Salvador Uribe-Carvajal, Arturo Cadena-Ramírez

**Affiliations:** 1 Departamento de Biología, Facultad de Química, Universidad Nacional Autónoma de México (U.N.A.M.), Ciudad de México 04510, Mexico; 2 Laboratorio de Bioprocesos Ambientales, Universidad Politécnica de Pachuca (U.P.P.), Zempoala, Pachuca 43830, Mexico; 3 Departamento de Microbiología e Inmunología, Facultad de Medicina Veterinaria y Zootecnia, Universidad Nacional Autónoma de México (U.N.A.M.), Ciudad de México 04510, Mexico; 4 Departamento de Bioquímica, Facultad de Medicina, Universidad Nacional Autónoma de México (U.N.A.M.), Ciudad de México 04510, Mexico; 5 Departamento de Farmacología, Instituto Nacional de Cardiología “Ignacio Chávez”, Ciudad de México 14080, Mexico; 6 Departamento de Bioterio, Instituto de Fisiología Celular, Universidad Nacional Autónoma de México (U.N.A.M.), Ciudad de México 04510, Mexico; 7 Departamento de Genética Molecular, Instituto de Fisiología Celular, Universidad Nacional Autónoma de México (U.N.A.M.), Ciudad de México 04510, Mexico

**Keywords:** nitrate reductase, Nar, new, method, activity, real-time, *Paracoccus denitrificans*, *Brucella*, denitrifiers, enteropathogenic

## Abstract

The transmembrane nitrate reductase (Nar) is the first enzyme in the dissimilatory alternate anaerobic nitrate respiratory chain in denitrifying bacteria. To date, there has been no real-time method to determine its specific activity embedded in its native membrane; here, we describe such a new method, which is useful with the inside-out membranes of *Paracoccus denitrificans* and other denitrifying bacteria. This new method takes advantage of the native coupling of the endogenous NADH dehydrogenase or Complex I with the reduction of nitrate by Nar through the quinone pool of the inner membranes of *P. denitrificans*. This is achieved under previously reached *anaerobic* conditions. Inner controls confirming the specific Nar activity determined by this new method were made by the total inhibition of the Nar enzyme by sodium azide and cyanide, well-known Nar inhibitors. The estimation of the Michaelis–Menten affinity of Nar for NO_3_^−^ using this so-called Nar-JJ assay gave a *K_m_* of 70.4 μM, similar to previously determined values. This new Nar-JJ assay is a suitable, low-cost, and reproducible method to determine in real-time the endogenous Nar activity not only in *P. denitrificans*, but in other denitrifying bacteria such as *Brucella canis*, and potentially in other entero-pathogenic bacteria.

## 1. Introduction

The nitrate reductase of the inner membranes of denitrifying bacteria or Nar is the central starting enzyme of the anaerobic alternative and dissimilatory denitrifying bacterial respiratory chains (see Supplementary Figure S1). It is a well-known enzyme in classic denitrifiers such as *Paracoccus denitrificans* [1,2,3]. The key role of Nar in *anaerobic* respiration has been shown physiologically, for instance, in some pathogenic enterobacteria by the non-virulent phenotype in ΔNAR knockout mutants together with null mutations in the other assimilatory, water-soluble, and/or periplasmic nitrate reductases (Nap) [4,5]. Since the gut is practically anaerobic, these nitrate reductases are essential enzymes for the colonization and virulence of pathogenic enterobacteria; thus, nitrate reductases are acquiring more clinical or biomedical interest. Furthermore, the additional feature of the membranous and dissimilatory nitrate reductase or Nar is that it is one of the elite bioenergetic enzymes that are capable of contributing to the transmembrane proton gradients since it is the only nitrate reductase that works as a proton pump coupled to nitrate reduction, and that can be used for ATP synthesis [1] (see Supplementary Figure S1). Given its bioenergetic and physiological relevance, in this work, we will focus on the membrane dissimilatory nitrate reductase or Nar to describe a useful new real-time method to measure the specific Nar activity, without detecting the other cytosolic or periplasmic nitrate reductases (Nap) (see the center of Supplementary Figure S1).

**Figure 1:**
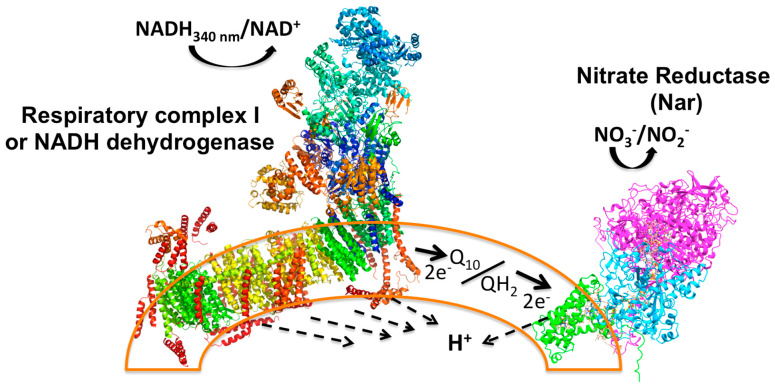
Principle of the Nar-JJ coupled assay method. The left side shows the Complex I structure (PDB_ID 4UQ8) assembled in the inner plasma membrane of SBP (sub-bacterial particles), shown as a partial arc (orange transparent bilayer) of a full SBP, simulating a fragment of a pseudo-spherical inverted inner plasma membrane. NADH works as the electron donor that is oxidized by Complex I to NAD^+^ (+H^+^, see Equations (1)–(3)), and this reaction is coupled to proton transport across the membrane to the internal aqueous phase of the SBP (dashed arrows). Two electrons from NADH are transferred through the electron transfer prosthetic groups of Complex I to the quinone pool (Q_10_) to produce fully reduced quinol (QH_2_) (see dark thick arrow). The quinone pool works as an electron transfer intermediate between Complex I and the Nar enzyme. Two electrons are then transferred to Nar to reduce NO_3_^−^ to NO_2_^−^ with the formation of a water molecule, and scalar protons (see Equations (1)–(3)). This reaction is also coupled to the transfer of protons across the membrane to the inner aqueous space of SBP. No other respiratory complexes participate due to the *anaerobic* conditions and the inhibition of Complex II or succinate dehydrogenase by malonate. See text for further details.

Although in *P. denitrificans* and other bacteria, the denitrifying chain is expressed preferably under *anaerobic* conditions [6,7], the quinone pool is kept reduced as QH_2_ by the respiratory Complex I and Complex II of the oxygenic respiratory chain, (see Supplementary Figure S1). Thus, in *P. denitrificans* and in other eubacteria, the full redox loop of the Nar enzyme in vivo is composed by CI or CII, the quinone pool, and the Nar enzyme, particularly under *anaerobic* conditions where the denitrifying chain is strongly expressed (see left side of Supplementary Figure S1). The method designed here takes advantage of this native redox loop and determines in real-time the rate of NADH oxidation by CI coupled to the reduction of nitrate by Nar under *anaerobic* conditions, where the rest of the oxygenic respiratory chain (succinate dehydrogenase or Complex II, *bc*_1_ or Complex III, Cytochrome *c*, and Cytochrome-*c* oxidase or Complex IV) is inactive because of the absence of oxygen (see Figure 1). In in vivo experiments, once nitrate is reduced and nitrite is formed, it can be further reduced to nitric oxide (NO) by the periplasmic nitrite reductase (Nir), and subsequently, NO can be further reduced by the membranous nitric oxide reductase (Nor) to nitrous oxide (N_2_O), and the latter can be reduced by the periplasmic N_2_O reductase (Nos) to molecular dinitrogen (N_2_) and water [8] (see right side of Supplementary Figure S1). The method proposed here is designed to measure only the specific activity of the first denitrifying reaction of the nitrate reduction by the membrane-embedded Nar, but not the following denitrifying reactions.

Functionally, to reduce one nitrate molecule (NO_3_^−^) to nitrite (NO_2_^−^), Nar receives two electrons (2e^−^) from the quinol at the positive side of the membrane (P-side) and these are transferred to the two *b*-type hemes, and ultimately transferred to the site of the nitrate reduction at the catalytic α subunit that coordinates the Mo atom by two sulfur atoms provided by two cofactor molecules (MGD) at the negative side of the membrane (N-side) (see Supplementary Figures S1 and S2 and Figure 1). Meanwhile, two vectorial protons (H^+V^) from quinol are released to the periplasm (P-side) (see Supplementary Figure S1 and Figure 1), and two more scalar protons (H^+S^) are taken from the N-side to form a molecule of water [8]. In the coupled CI/Nar reaction, the net result is a molecule of NADH that fully reduces Q_10_ to quinol, which becomes fully oxidized, and a molecule of nitrate (NO_3_^−^) that is fully reduced to nitrite (NO_2_^−^), following the overall reaction, including the NADH dehydrogenase (see also Figure 1):

NADH + Q_10_ ⇔ NAD^+^ + QH_2_ +H^+S^ + 4H^+V^
(1)
QH_2_ + NO_3_^−^ +2H^+S^ ⇔ NO_2_^−^+H_2_O + Q_10_ + 2H^+V^

(2)
NADH +NO_3_^−^ ⇔ NAD^+^ +NO_2_^−^ +H_2_O +H^+S^ + 6H^+V^

(3)

The arrows show reversibility; however, in the Nar-JJ assays, NADH and NO_3_^−^ are added at relatively high concentrations so the net reactions are flowing to the right side to the oxidation of NADH and reduction of NO_3_^−^ to NO_2_^−^.

Although the Nar enzyme is well-known at the atomic structural level [9], the functional characterization of the enzyme has been limited to a few activity determinations of the membrane-extracted and isolated Nar by discontinuous or continuous methods, with the latter using non-physiological substrates [2]. In the discontinuous methods, the residual nitrate [NO_3_^−^], or the appearing nitrite [NO_2_^−^] concentrations, are the ones determined by complicated protocols. The latter rely on several blank controls to discard interferences by the substances present in the samples; thus, these additional controls decrease their accuracy [10]. On the other hand, in the reported continuous methods (or in real-time), the isolated Nar enzyme has been followed under *anaerobic* conditions in the presence of artificial electron donors, substrates, or exogenous enzymes [2]. For the latter continuous procedures, only brief procedures have been described in reports characterizing the purified Nar [2], or applied only to cytoplasmic bacterial extracts [11]. Thus, the latter measures the soluble Nap, other cytosolic nitrate reductases, or NADH oxidases, but not the membranous Nar. For instance, the latter procedure shows a strong basal NADH consumption without NO_3_^−^ addition, likely derived from other soluble NADH oxidases. In this case, the subtraction of a high basal NADH decay could lead to serious signal-to-noise errors and uncertainties. Finally, there is another Complex I/Nar assay that has been reported in previous research, similar to the one detailed here; however, there is no full description of the method, but only a brief mention that it was made in anaerobic conditions obtained by the N_2_ flow in the reaction cells. Furthermore, the authors explain that they did not validate this method by confirming whether the Nar turnover involved the rate-limiting step of the fully coupled Complex I-Nar reaction, so they indicate that their kinetic determinations of Nar activity could be limited in accuracy [12]. As will be discussed later, in this work, we confirmed clearly that the Nar turnover is by far the slower reaction taking place; thus, Nar the rate-limiting enzyme in the coupled Complex I/Nar reaction. Therefore, this new Complex I/Nar assay is fully validated in this work. 

The new method introduced here to determine the endogenous Nar activity of the inside-out membranes of *P. denitrificans* and of other denitrifying bacteria, is thoroughly detailed for its generalized application. It consists of a real-time simple coupled spectrophotometric assay where the 1:1 stoichiometric NADH/NO_3_^−^ oxidation/reduction is preserved because the respiratory Complex I (CI) is kinetically and thermodynamically coupled to the reduction of nitrate by the Nar enzyme under *anaerobic* conditions (see Figure 1). The latter *anaerobic* conditions are achieved through oxygen exhaustion by respiratory Complex II or succinate dehydrogenase, followed by its inhibition by malonate. Finally, once the basal NADH decay is stabilized and nearly zero, the Nar reaction is started by NO_3_^−^ addition under the previously achieved *anaerobic* conditions. The Nar-specific activity of the inside-out membranes or sub-bacterial particles (SBP) of *P. denitrificans* and other denitrifying bacteria is obtained by the subtraction of the near-zero basal NADH oxidation from the faster slope of the NADH decay after the addition of NO_3_^−^. One can calculate the specific Nar activity in SI units (μmol/(min.•mg.pt.)) from the rate of the NADH decay given the 1:1 stoichiometry of oxidized NADH per reduced NO_3_^−^. This so-called Nar-JJ method is specific for the endogenous Nar enzyme of SBP, as shown by the appropriate specific inhibition with sodium azide and is suitable to determine the endogenous Nar activity in real-time with its natural substrates. The advantages of this method over the previous discontinuous or non-natural continuous methods are that this is a simpler, cheaper, continuous, real-time, reliable, and reproducible method, which uses natural substrates, and can be used straightforwardly. This Nar-JJ method could be applied to compare, for instance, the Nar activity of SBP obtained from denitrifying bacteria grown under different conditions, or between different innocuous or pathological bacterial species or strains. Future applications in biotechnology and the environmental and clinical microbiology of denitrifying bacteria will be suggested and discussed below.

### 1.1. Objective 

The objective of this paper is to contribute a new method to determine in real-time the nitrate reductase (Nar) specific activity in inverted membranes or sub-bacterial particles (SBPs) of Nar-expressing denitrifying bacteria including *Paracoccus denitrificans* and *Brucella canis*.

### 1.2. Significance

The nitrate reductase or Nar enzyme is the starting and key enzyme of the alternate dissimilatory *anaerobic* respiratory chain of several denitrifying bacteria. In addition to being expressed and characterized in *P. denitrificans*, it is also an essential enzyme for the virulence of several entero-pathogenic bacteria that colonize the intestine in nearly *anaerobic* conditions. This has been demonstrated by Nar knockouts where the deletions of Nar and other nitrate reductases in pathogenic *E. coli* or *Salmonella enterica* lead to the loss of the virulent properties of these bacterial pathogens [4,5]. Here, we contribute with a relatively simple, real-time, low-cost, reproducible, and suitable method to determine the specific activity of the Nar enzyme embedded in its native bacterial inner plasma membranes of inverted inside-out vesicles or sub-bacterial particles (SBP). This improves the previous methods to measure Nar activity since it uses its natural endogenous redox substrates. Here, we also demonstrated that this Nar-JJ method can be applied to the entero-pathogenic α-proteobacterium *Brucella canis*, thus contributing with a new methodology to assess the effectiveness of new antibacterial agents targeting the nitrate reductase or Nar enzyme of this or other bacterial pathogens. This is of particular significance given the worldwide threat of bacterial multi-resistance to the currently available antibiotics. Additionally, this new Nar-JJ method can be used to assess the Nar activity and denitrifying capacity in *P. denitrificans* and other denitrifying species and strains used in water-treatment plants or in the food industry to remove nitrate or nitrite from waste water or drinking water, or from commercially available foods or drinks.

## 2. Results and Discussion

We have a custom procedure to obtain SBP from *P. denitrificans* by osmotic shock, differential centrifugation, and ultracentrifugation (see Section 3); this procedure can produce highly coupled inside-out vesicles that show, for instance, the highest rates of coupled ATP synthase activity as driven by succinate or NADH oxidation [13,14,15]. This tight membrane coupling is essential for coupled ATP synthesis, but not for the Nar-specific activity determined here, since the coupling achieved between NADH dehydrogenase or Complex I and the Nar enzyme takes place kinetically and thermodynamically through the quinone (Q_10_) pool, and not through the proton motive force (Δμ_H+_), as occurs with the OxPhos reaction (see Supplementary Figure S1). Nevertheless, the SBP were always incubated and used in appropriate osmotic media to maintain the integrity of the SBP membranes through all procedures. The careful process of this new in vitro real-time method for the determination of Nar-specific activity has some closer similarities to the in vivo Nar reaction method than the previous procedures using non-natural substrates.

### 2.1. Obtaining the Anaerobic Conditions in the Reaction Cells before Starting the Nar Reaction

**Figure 2:**
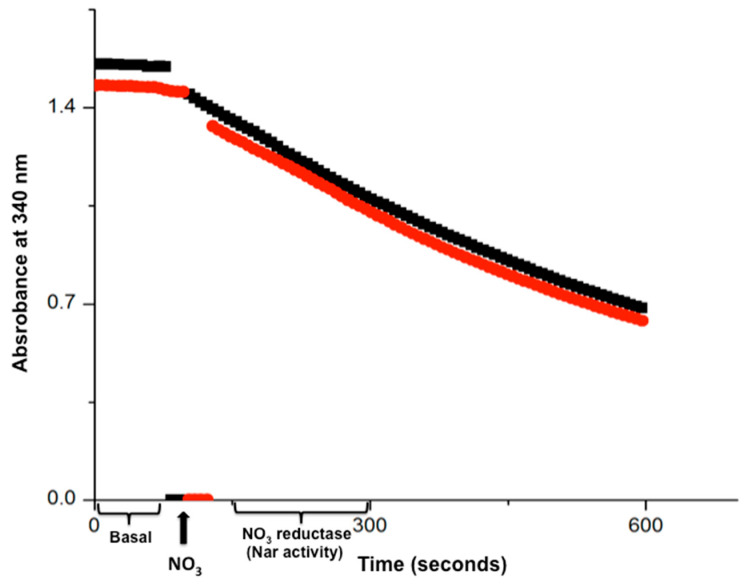
The time course of the real-time Nar-JJ assay carried out with SBP from *P. denitrificans*. The *anaerobic* reaction cells were totally filled with SBP from *P. denitrificans* included, and previously depleted of dissolved oxygen by 15 min pre-incubation of succinate respiration. Once oxygen was depleted, 20 mM of malonate was injected to inhibit further Complex II or succinate dehydrogenase activity. This ensures that all electrons used to reduce NO_3_^−^ by Nar come exclusively from NADH. The nearly zero basal initial slope starting at zero time was read after adding NADH and before the addition of NO_3_^−^, and this slow NADH decay is used as basal activity to be subtracted from the coupled NADH/NO_3_^−^ oxidation-reduction carried out by Complex I and the Nar enzyme, after the addition of NO_3_^−^ (black thick arrow, bottom). The most linear portion of each trace is used to determine the specific Nar activity of SBP. Red and black traces show duplicates. A representative duplicate of at least 4 different assay determinations is shown.

In the first successful real-time Nar assays, we pre-incubated 300 μg of SBP for 10 min at 37 °C in sealed and filled 2 mL anaerobic cells with “Nar reconstitution buffer” so the oxygen dissolved in the 2 mL cells was consumed in the first 5–10 min. However, we extended this pre-incubation time to 15 min to ensure the maximal oxygen depletion within the filled reaction cells (the total depletion of oxygen in these assays was demonstrated by Oroboros oximetry (see below)). From that point on, further additions to the *anaerobic* cells were carried out by a microinjection of the minimal volume (1–20 μL) of highly concentrated stocks, so that the injection of dissolved oxygen into the already *anaerobic* cells was minimized. Afterward, 20 mM of malonate was added from a 4 M stock to completely inhibit the succinate dehydrogenase or Complex II. This was to avoid any further reduction in nitrate by succinate, so this will ensure that all the electrons reducing the nitrate were derived only from NADH and transferred by Complex I to maintain the 1:1 NADH/NO_3_^−^ stoichiometry in this procedure. At this point, the absorbance at 340 nm of the *anaerobic* cells were read at 37 °C to check that the basal NADH decay was near zero (see Figure 2). 

We found that in some SBP preparations, this was not the case; thus, the basal NADH decay slope was higher, for instance when working with *anaerobically* grown cells of *P. denitrificans* where the alternate oxidases were overexpressed [8]. Since these alternate oxidases have higher affinities for oxygen than Complex IV, they could consume NADH at very low oxygen concentrations, thus explaining the higher basal NADH decays obtained with *anaerobically* grown *P. denitrificans* SBP. In contrast, when these bacteria were grown *aerobically* in respiratory media, then the basal NADH consumption was near zero after the 15 min of succinate oxidation. With this rationale, to avoid the higher basal NADH consumption after the depletion of oxygen by succinate, we added glucose oxidase + glucose to scavenge the scarce oxygen present in the *anaerobic* cells. We added 5 μL of a concentrated (20 mg/mL) glucose oxidase stock to the *anaerobic* cells containing 10 mM glucose; this enzyme was added after inhibiting CII with malonate, stirred, and pre-incubated for a further 5 min before adding the NADH, and when that was complete, the basal activity returned to near zero within the 15 min of succinate respiration plus the 5 min of glucose oxidase addition in essentially all cases (see below and Supplementary Figure S3). 

**Figure 3:**
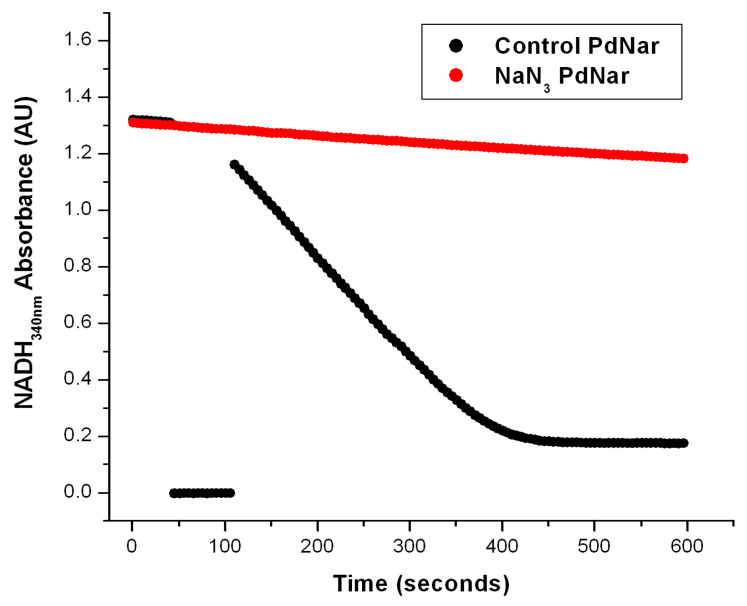
The effect of NaN_3_ on the Nar-specific activity of SBP from *P. denitrificans*. The anaerobic reaction cells were prepared as described in Figure 2 and in the Section 3. In the black trace, the basal activity was determined from zero time on the first seconds of reaction before the first 50 s, when the addition of NO_3_^−^ was carried out as described before. The most linear segment of the negative slope was used to calculate the Nar-specific activity. The red trace shows the Nar activity determined with SBP from *P. denitrificans* but pre-incubated in the presence of 5 mM of NaN_3_ after the addition of 20 mM of malonate. In this case, the nitrate (NO_3_^−^) was added before time zero (t ≤ 0) from the very beginning. As shown, the basal activity of the control trace (black) was very similar to the red trace in the presence of 5 mM of NaN_3_. Therefore, the net Nar activity with 5 mM of NaN_3_ was essentially zero.

**Figure 4:**
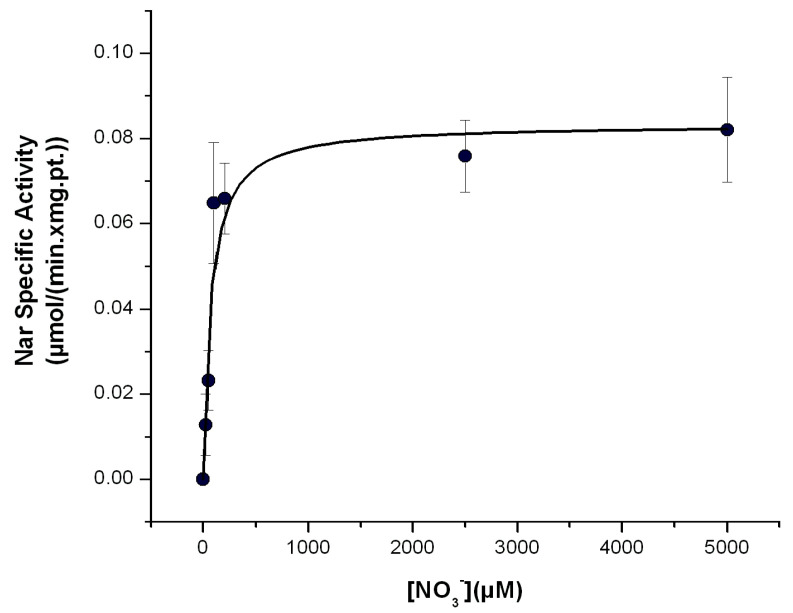
Determination of the Michaelis–Menten affinity (*K_m_*) of the Nar enzyme for nitrate with the Nar-JJ assay. The CI/Nar coupled reactions were determined at the different concentrations of nitrate ([NO_3_^−^]) shown, in *anaerobic* cells, as described in the Section 3, at 37 °C. Three independent determinations were averaged, and the standard error (±S.E.) bars are shown for each data plot. The data points were fitted by non-linear regression to the Michaelis–Menten equation in the Origin 7.0 Software and the kinetic values obtained are as follows: *V_max_* = 83 ± 7 nmol/(min.•mg.pt.) and *K_m_* = 70.4 ± 24.7 μM. This value is in the μM range, in concordance with previous determinations of the *K_m_* for NO_3_^−^, using natural substrates of the Nar enzyme. See text for further details.

When the basal NADH consumption was near zero, we were confident that most of the dissolved oxygen was already consumed and that the conditions to start the coupled Complex I/Nar NO_3_^−^ reductase reaction were optimal. After adding 5 mM of KNO_3_^−^, we found an accelerated NADH decay coupled to the nitrate reduction in the first 5–10 min of real-time monitoring spectrophotometrically at 340 nm and 37° C (see Figure 2 and Figure 3). Several NO_3_^−^ concentrations were assayed and the optimal NADH decay rate was obtained with 5 mM of NO_3_^−^, which was a saturating concentration (see Figure 4). From that point on, all assays shown contained this NO_3_^−^ concentration of 5 mM, except for the experiments shown in Figure 4.

To confirm that this NADH decay observed in the *anaerobic* reaction cells was specific for its oxidation by the Nar enzyme, and not by the Nap one, we took advantage of the specific inhibition of Nar by sodium azide, versus its non-inhibition of the Nap enzyme by NaN_3_; this has been well-characterized before [2,16]. We thus expected a total inhibition by azide of the *anaerobic* oxidation of NADH by NO_3_^−^ catalyzed by the Nar enzyme; any residual *anaerobic* NADH oxidation would mean the presence of the Nap enzyme or of other NADH oxidases. As can be seen in Figure 3, the addition of 5 mM of NaN_3_ produced a total inhibition of the *anaerobic* oxidation of NADH by NO_3_^−^, and this confirmed that the observed NADH decay in these conditions is associated exclusively to the Nar activity, and there is essentially no contamination by Nap or other NADH oxidases. This is likely because these Nar-JJ assays are carried out with highly purified SBP lacking cytoplasmic and periplasmic enzymes. In addition to this, we added 5 mM of potassium cyanide (KCN) to a separate reaction mixture in *anaerobic* cells, and this inhibitor also produced a total inhibition of the *anaerobic* oxidation of NADH by NO_3_^−^. This was not unexpected since the structure of the Nar enzyme is relatively similar and related to the Cytochrome-*c* oxidase or Complex IV family of enzymes, thus having a high sensitivity to KCN [2,8,16]. Thus, taken together, these inhibition studies clearly confirm that the *anaerobic* oxidative NADH decay observed under our actual conditions derives exclusively from the Nar enzyme, and not from the Nap nitrate reductase or from other NADH oxidases putatively present in the SBP preparations.

As a last control of this Nar-JJ assay, we also conducted the *anaerobic* determination of the Nar activity in SBP prepared from the wild type *P. denitrificans* Pd1222 strain grown in two different conditions; one SBP preparation was obtained from the *aerobic* growth of Pd1222 in succinate media, and another SBP preparation was obtained from Pd1222 grown under *anaerobic* conditions in nitrate media, as described in the Section 3. 

It is well-known that the Nar enzyme and the rest of the denitrifying respiratory chain are overexpressed under *anaerobic* conditions in *P. denitrificans*, whereas in respiratory *aerobic* conditions, the Nar enzyme and the accompanying denitrifying complexes are present in lower amounts; therefore, the denitrifying activity is controlled to be expressed preferably in low oxygen amounts or in its absence [7]. Accordingly, we expected a higher Nar activity in SBP isolated from Pd1222 grown under *anaerobic* conditions than the Nar activity of SBP isolated from Pd1222 grown in *aerobic* conditions.

The comparison of the Nar activity assays clearly showed that the Nar activity obtained from SBP prepared from Pd1222 grown under *anaerobic* conditions, as determined by the present Nar-JJ assay, increased to 105 ± 24 nmol/(min.•mg.pt.) (±S.E.) after four independent determinations (see a representative experiment in Figure 3, black trace). In contrast, the Nar specific activity of the *aerobically* grown cells of *P. denitrificans* was 43 ± 6 nmol/(min.•mg.pt.) (±S.E) after four independent determinations (see a representative duplicate experiment in Figure 2). Therefore, the Nar specific activity was about 2.5-fold higher in SBP isolated from the Pd1222 strain grown in *anaerobic* conditions, than the Nar activity obtained in SBP isolated from the same cells grown in *aerobic* conditions (compare the faster “*anaerobic*” black trace of Figure 3 with the slower “*aerobic*” duplicate red and black traces of Figure 2). This is consistent with a higher expression of the Nar enzyme, and the rest of the denitrifying electron transport chain, in the *anaerobically* grown *P. denitrificans* cells, and thus a higher Nar presence in the SBP isolated from the *anaerobically* grown bacteria, as demonstrated by proteomic analyses [7]. These results confirm the specificity of this Nar assay, i.e., that this method does not determine any other nitrate reductase activity but specifically the Nar activity, and that the real time NADH decay observed in this method is in good agreement with the higher expression of Nar, and of the rest of the denitrifying alternate respiratory chain of *P. denitrificans*, under *anaerobic* conditions, vs. its lower expression in *aerobic* growth conditions.

### 2.2. Kinetic Validation of the NADH Dehydrogenase or Complex I Activity as the Coupling Reaction for the NO_3_^−^ Reduction by Nar in SBP

Formally, to accept any coupling reaction in a kinetically coupled enzymatic assay system, the coupling enzyme or enzymes should be present in at least 5–10-fold kinetic excess in total specific activity than the target enzyme to be determined. This is to leave the target enzyme as the rate-limiting step of the full coupled reactions, thus avoiding a case where the coupling enzyme or enzymes would be rate-limiting. To validate the endogenous specific activity of the NADH dehydrogenase or Complex I as a suitable coupling enzyme for Nar, we determined the rate of NADH dehydrogenase in SBP spectrophotometrically under exactly the same conditions used for the Nar determination, with the exception that this was carried out under *aerobic* conditions, i.e., without oxygen depletion by succinate pre-incubation, and with the reaction cells fully open to the air. This was carried out to determine the maximal specific rate of Complex I by adding NADH (0.2 mM) in the absence of any respiratory chain inhibitor. This was carried out separately with the SBP isolated from both *aerobic* and *anaerobic* conditions of the wild-type Pd1222 strain, and compared with similar NADH dehydrogenase rates reported by others [17]. Accordingly, we observed a rate of NADH dehydrogenase-specific activity in SBP of 1.4 μmol/(min.•mg.pt.) with the same activity in SBP isolated from *aerobically* or *anaerobically* grown *P. denitrificans* cells. Coincidentally, the previously reported rate of NADH dehydrogenase, measured as the NADH:Q_2_ reaction with SBP prepared from *P. denitrificans*, was 1.7 μmol/(min.•mg.pt.) [17]. It is worth recalling that this NADH:Q_2_ reaction is the actual coupling reaction taking place in this proposed Nar-JJ assay, since Nar oxidizes the quinol reduced by Complex I (see Equations (1)–(3) and Figure 1). Therefore, the maximal specific NADH:Q_2_ activity of the CI in SBP prepared from *P. denitrificans* is 39-fold higher than the Nar-specific activity in SBP prepared from *aerobically* grown *P. denitrificans* cells (CI/Nar = 1.7/0.043 = 39), and 16-fold higher than the Nar-specific activity of SBP prepared from *anaerobically* grown *P. denitrificans* cells (CI/Nar = 1.7/0.105 = 16). Taken together, these results show that for the determination of an accurate endogenous Nar-specific activity, the endogenous NADH dehydrogenase or Complex I is not the rate-limiting step of the coupled CI/Nar reaction, but it is indeed the limiting Nar-catalyzed NO_3_^−^ reduction to NO_2_^−^; thus, Complex I can be optimally used as the coupling enzyme for this Nar-JJ assay in SBP prepared from *aerobically* or *anaerobically* grown *P. denitrificans* cells. This is consistent with the constitutive expression of the classical or “mitochondrial-like” full respiratory chain of *P. denitrificans* in *aerobic* or *anaerobic* conditions, where it was observed that the respiratory complexes I–IV, including the complex V or ATP synthase, are expressed in very similar amounts either in *aerobic* or *anaerobic* conditions [6,7]. This is in contrast with the alternate denitrifying chain starting with the Nar enzyme, which is overexpressed in *anaerobic* conditions vs. its lower expression in *aerobic* conditions [6,7]. This proper kinetic coupling of Complex I to the Nar enzyme in SBP of *P. denitrifcans* makes this proposed Nar-JJ method a relatively low-cost protocol, since it does not require the addition of any additional exogenous coupling enzymes to work properly.

### 2.3. Estimation of the Affinity of Nar for NO_3_^−^ with the Nar-JJ Method

The affinity of Nar for its substrate NO_3_^−^ has been estimated by the previously mentioned indirect and non-natural substrate methods, and in some instances, the affinity goes to the 1–400 μM range, but in other estimations it goes up to 1.5 mM [2,16]. It has been discussed that the latter mM estimations could arise from the use of non-natural substrates as electron donors such as methyl viologen or others. When the *K_m_* for NO_3_^−^ is estimated with the Nar natural substrates as reduced quinols, the determined *K_m_* values go down to the μM range for the *P. denitrificans* Nar [2,3,12] and also for the homologous *E. coli* Nar [18]. Therefore, using natural substrates is a condition that is closer to the in vivo conditions in the bacterial cell membrane than using non-natural substrates; thus, it seems that a value in the μM range for the *K_m_* of NO_3_^−^ is closer to the bacterial physiological conditions than the mM *K_m_* for NO_3_^−^ estimated using non-natural electron donors [3]. One of the advantages of this Nar-JJ assay is that it was designed to simulate more or less closely the bacterial in vivo environment of growth under *anaerobic* conditions by estimating the Nar-specific activity with the natural substrates and the NADH dehydrogenase-QH_2_-Nar redox loop in its natural inner bacterial cell membrane and under nearly *anaerobic* reaction cells. Therefore, it is expected that estimations of the *K_m_* for NO_3_^−^ should be closer to the μM range, as shown when the Nar activity has been estimated using natural electron donors [2,3,12,18]. To determine the *K_m_* for NO_3_^−^ by the Nar-JJ method, we repeated the same protocol for the Nar determination with SBP under *anaerobic* conditions but changed the [NO_3_^−^] concentrations in the μM to mM range. We carried out these determinations with the SBP prepared from *P. denitrificans* Pd1222 cells grown under *anaerobic* conditions, since, as shown before, these ones catalyzed the Nar NO_3_^−^ reduction at the highest specific activity rates. We confirmed the near zero slope in the basal NADH consumption before the addition of NO_3_^−^ in all [NO_3_^−^] concentrations used and proceeded to estimate the Nar-specific activity by subtracting the basal rate of NADH decay before the addition of NO_3_^−^ to the faster negative slopes of NADH decay after addition of [NO_3_^−^]. We obtained, after three independent determinations, clear hyperbolic Michaelis–Menten kinetics, and adjusted the plot accordingly with a non-linear curve fit kinetic analysis in Origin 7.0, obtaining an estimated *K_m_* of 70.4 ± 24.7 μM which is in the μM range, as expected from the estimation using natural substrates [2,3,12,18] (see below). 

In agreement with previous determinations, it has been reported that in inverted membranes of *P. denitrificans*, using a similar CI/Nar coupled assay but injecting N_2_ to obtain anaerobic conditions, the *K_m_* for NO_3_^−^ obtained was in the range of 40–75 μM [12]. It is worth noting that the authors that obtained these previous estimations warned that they did not confirm whether the Nar reaction was or not the rate-limiting step of their CI/Nar coupled assay; so, the authors indicate that their kinetic Nar data should be taken with caution [12]. In this work, we clearly confirmed that the NO_3_^−^ reduction by Nar is by far the slowest reaction of the CI/Nar-coupled reaction, of course assuming that the QH_2_ diffusion between Complex I and Nar is not limiting, as suggested by the fact that the quinone pool is in a molar excess by far, compared to the amounts of respiratory complexes in these energy transduction membranes, including Complex I and Nar [8]. In this context, we could thus infer that the previous data obtained with inverted membranes of *P. denitrificans* [12] were acquired in conditions where the NO_3_^−^ reduction by Nar was indeed the rate-limiting step; therefore, these previous determinations of the *K_m_* of Nar for NO_3_^−^ in inverted membranes of *P. denitrificans* (40–75 μM) are accurate and thus agree very well with our own Nar NO_3_^−^ *K_m_* value of 70.4 ± 24.7 μM, determined here by the Nar-JJ assay. Taken together, our results and the discussed antecedents suggest that this estimated *K_m_* of Nar for NO_3_^−^ is a close estimation of the affinity of the Nar enzyme for NO_3_^−^ under the native or in vivo growth conditions of *P. denitrificans*, with the Nar enzyme embedded in its natural inner bacterial membrane. Nevertheless, our *anaerobic* in vitro conditions will always be different to the actual in vivo bacterial cytoplasm. In the whole *P. denitrificans* cells, it has been shown that the affinity of the Nar reaction for NO_3_^−^ goes down below 10 μM [12]; however, in this process with whole bacterial cells, the affinity of the NO_3_^−^ transporter(s) involved in the entrance of NO_3_^−^ into the bacterial cytoplasm should of course be reflected [12].

### 2.4. The Nar-JJ Assay Can by Applied to Other Denitrifying and Pathogenic Bacteria besides P. denitrificans

**Figure 5:**
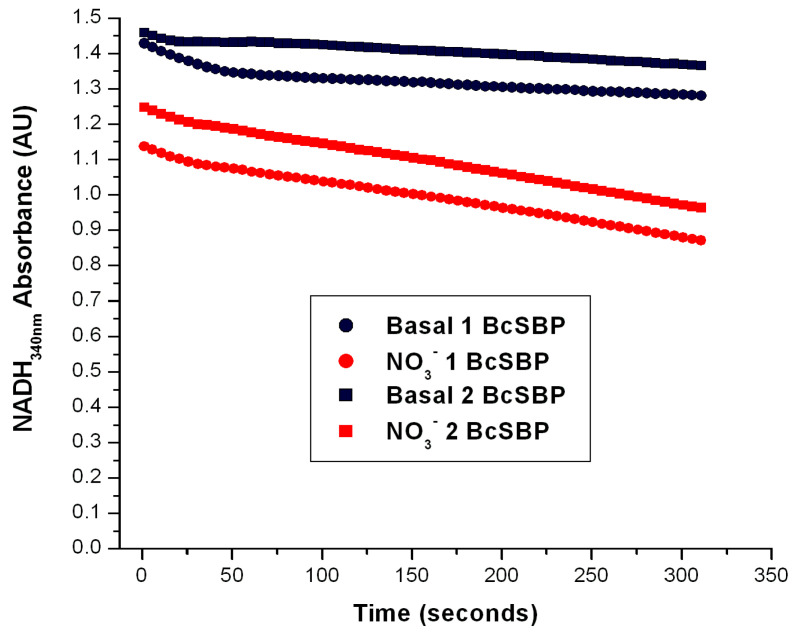
Determination of the Nar-specific activity in SBP from *Brucella canis*. The Nar-JJ assays were carried out with SBP prepared from *Brucella canis* grown under *aerobic* conditions as described for *P. denitrificans* in the Section 3. The red traces show a duplicate of the Nar-JJ assays carried out in *anaerobic* cells as described in the Section 3, starting the Nar reaction with 5 mM of NO_3_^−^ before the zero reading time (t ≤ 0). The black traces show the basal NADH oxidation in the absence of NO_3_^−^ during the full time course. The quick initial NADH decay in the first 50 s is due to residual oxygen, since these Nar assays were carried out in the absence of glucose oxidase and glucose, which scavenge residual oxygen and abolish these initial decay rates (see for instance Figure 4). The negative slope used to calculate the specific Nar activity of SBP from *B. canis* (BcSBP) was estimated between the 100 and 300 s of reaction to ensure full linearity of the Nar rate calculations. As can be seen, in *B. canis*, the Nar-JJ assay gives a reproducible linear NADH decay (red traces) that can be used to calculate the specific BcNar activity after subtraction of the basal NADH decay in the absence of NO_3_^−^ (black traces). See text for further details.

One of the possible limitations of this method would be that this could only be applied to *P. denitrificans* but not to other denitrifying bacteria. To assess if this Nar-JJ method could be applied to other denitrifying α-proteobacteria, we took advantage of membrane preparations available in our laboratory of the intracellular parasite *Brucella canis*, a member of the Brucella genus, the latter being responsible for the current worldwide health issue of Brucellosis in humans and some animals. It has been shown that the Nar enzyme is essential for the virulence of some pathogenic enterobacteria such as pathogenic strains of *E. coli* or *Salmonella enterica* [4,5], among others. Due to the nearly *anaerobic* conditions that are present in the gut, the members of the human and animal enteric microbiota have a bacterial physiology that is strongly dependent on *anaerobic* respiration by alternate respiratory chains [4,5]. Therefore, we assessed whether this Nar-JJ assay could be applied to the α-proteobacterial entero-pathogenic and intracellular parasite *Brucella canis*. We prepared SBP from inverted membranes of *B. canis*, with the same protocol as applied to the SBP prepared from *P. denitrificans* (see Methods) and obtained highly coupled inverted SBP from *B. canis* (BcSBP), which were used to determine the Nar activity in *anaerobic* cells with the same method and conditions used for *P. denitrificans* as described above. Since we grew *B. canis aerobically*, we observed that the Nar activity was similar to that observed for *P. denitrificans* SBP obtained from cells grown under *aerobic* conditions, but slightly slower, since it gave an average BcNar activity of 34 nmol/(min.•mg. pt.) (Figure 5), as compared to the 43 nmol/(min.•mg. pt.) obtained with PdSBP. This *B. canis* Nar activity (BcNar) was also inhibited to zero after the addition of 5 mM of NaN_3_, thus confirming that this method is also specific for the Nar enzyme from other bacteria and suitable to be applied to other denitrifying bacteria that possess both, the respiratory Complex I or NADH dehydrogenase and the Nar enzyme that is properly coupled in their inner membranes. 

This result opens a wide window of applications for this proposed Nar-JJ method with other denitrifying bacteria outside the α-proteobacterial class. For instance, in other entero-pathogenic bacteria such as pathogenic strains or species of *E. coli* and *Salmonella enterica*, among others containing both Complex I and the Nar enzyme that is properly coupled in their inner plasma membranes. This is relevant for clinical purposes, since the virulence of these enteric pathogens relies on the activity of the denitrifying respiratory chain [4,5], which is essential under the nearly *anaerobic* conditions of the animal and human internal light of the gut. We could also propose that this Nar-JJ method could be used in pathogenic bacteria where Complex I is absent and replaced by other NADH or NQR oxidases [19], such as the case of *Vibrio cholerae*, the virulent bacteria producing cholera, provided that the proper and 1:1 stoichiometric coupling of the alternate NADH and/or NQR oxidases to the nitrate reduction by Nar could be demonstrated.

In summary, this Nar-JJ method can be applied to other denitrifying and pathogenic bacteria, provided that it has been previously confirmed that the Complex I or the respective alternate NADH dehydrogenase(s) are not the rate-limiting step of the respective NADH dehydrogenase–QH_2_–Nar redox loop, but that Nar is the limiting one, and that a 1:1 stoichiometry can be ensured between the oxidized NADH and the reduced nitrate. 

With these criteria fulfilled, this Nar-JJ method can be used, for instance, in the search for novel antimicrobials or antibiotics targeting the Nar enzyme of denitrifying bacterial pathogens, confirming that the proposed antimicrobials do not affect the activity of Complex I or of the respective coupled NADH dehydrogenase(s) of the bacterial pathogens studied. On the other hand, this method can be used during other biotechnological applications where denitrifying bacteria are used. For instance, in water-treatment plants, or any other biotechnological application where the denitrification activity of *P. denitrificans* is essential. Although *P. denitrificans* is always present in water-treatment plants as the major denitrifier, other denitrifying bacteria are also present; so, the determination of the Nar activity is important to evaluate the denitrifying capacity of the bacterial strains used in these or other biotechnological applications.

There is an alternative way to obtain the desired *anaerobic* conditions to start the Nar reaction, in addition to our procedure of exhausting the dissolved oxygen within the sealed *anaerobic* cells by respiration with succinate, and then inhibiting Complex II with malonate. This has been applied before in a similar CI/NO_3_^−^ coupled assay method [12], and it consists of injecting a controlled N_2_ current into the reaction cell at the upper surface of the aqueous phase for several minutes to remove the dissolved oxygen in the reaction media. Although this method seems useful, we preferred the oxygen depletion by succinate respiration to avoid the formation of bubbles that interfere strongly with absorbance readings, but if this bubble formation could be avoided somehow, this could simplify the present Nar-JJ method by removing the actual oxygen depletion step by succinate respiration and its inhibition by malonate. If this can be properly accomplished, the coupled CI/Nar reaction could be started by the NADH addition and basal reading determination confirming the near-zero slope, followed by the addition of NO_3_^−^ to start the Nar reaction. 

As carried out before in a similar assay in *P. denitrificans* inverted membranes [12], we also assessed the effect of uncoupling the SBP membranes with the protonophore FCCP (3–5 μM), in case the vectorial protons pumped by Complex I and by Nar towards the inner aqueous space of the SBP (see Figure 1) could slow down the CI/Nar coupling or this “forward” electron transfer; however, we did not observe any significant effect of FCCP on the Nar-specific activity of PdSBP. This could be probably due to an inner proton leakiness of the SBP membranes, or to the requirement of a higher proton gradient than the one formed in SBP under the achieved *anaerobic* conditions, to reverse the electron flow through the redox NADH–quinone–Nar loop in this coupled Nar-JJ assay. However, if desired, FCCP or another proton uncoupler could be added to the reaction media to ensure that the proton backflow through Complex I or Nar will not slow down the Nar nitrate reduction of SBP under these conditions.

### 2.5. Depletion of Dissolved Oxygen in the Anaerobic Reaction Cells of the Nar-JJ Method

As mentioned in the Section 3, although we use the terms “*anaerobic*”, “*anaerobically*”, or “*nearly anaerobic*” as synonyms, we did not determine the exact amount of residual oxygen dissolved in the bacterial growth media. However, in similar growing *anaerobic* media and conditions scaled up to a 4 L Biorreactor, by flowing CO_2_ we kept the amount of residual oxygen in the media below 5% during the progress of the growth curve of *P. denitrificans* in *anaerobic* conditions (Sharon Rojas-Alcantar and José J. García-Trejo, unpublished observations). However, rather than the residual oxygen in the growth media, a more crucial variable was the amount of residual oxygen dissolved within the spectrophotometric *anaerobic* reaction cells during the Nar-JJ assays. The oxygen depletion step of succinate respiration before the addition of nitrate in the Nar-JJ assays (see Section 3) lasts 15 min in the case of SBP isolated from *P. denitrificans* grown *aerobically*, and a further 5 min step of glucose oxidase + glucose was added to scavenge the traces of residual oxygen when SBP were isolated from *P. denitrificans* cells grown *anaerobically*, thus giving a total of 20 min of the oxygen depletion step in SBP isolated from *anaerobically* grown Pd1222 cells. The custom *anaerobic* spectrophotometric cells were about 2 mL in total volume. We assumed from previous oxidative phosphorylation and respiration experiments with similar SBP preparations, that the oxygen was totally consumed during these 15–20 min of the oxygen depletion step by succinate respiration. This oxygen depletion was evidenced by the near-zero NADH basal consumption after the succinate respiration step and malonate addition (see Figure 2, Figure 3 and Figure 5). However, to confirm that the oxygen was totally consumed with both SBP preparations, we had to determine the final amount of residual dissolved oxygen during the 15–20 min of the succinate respiration step. To achieve this, we had to measure the rate of oxygen consumption within the sealed *anaerobic* cells in a similar 2 mL volume, using exactly the same experimental conditions of the Nar-JJ assay, with the same buffer and assay composition, including SBP at the protein concentration used and starting the respiration with the custom succinate concentration. The closest oximeter closed cells of 2 mL that were more similar to the *anaerobic* spectrophotometer cells used for the Nar-JJ assays were those of the Oroboros oxygraph. Thus, we reproduced the oxygen depletion step of succinate respiration with exactly the same buffer and experimental conditions used for the Nar-JJ assays, but in the Oroboros 2 mL closed cells instead of the spectrophotometric *anaerobic* cells. We used exactly the same temperature (37 °C), buffer, and SBP concentrations in both preparations, i.e., those isolated from *P. denitrificans* grown *aerobically* in one of the Oroboros cells, and those isolated from *P. denitrificans* grown *anaerobically* in the other cell. 

**Figure 6:**
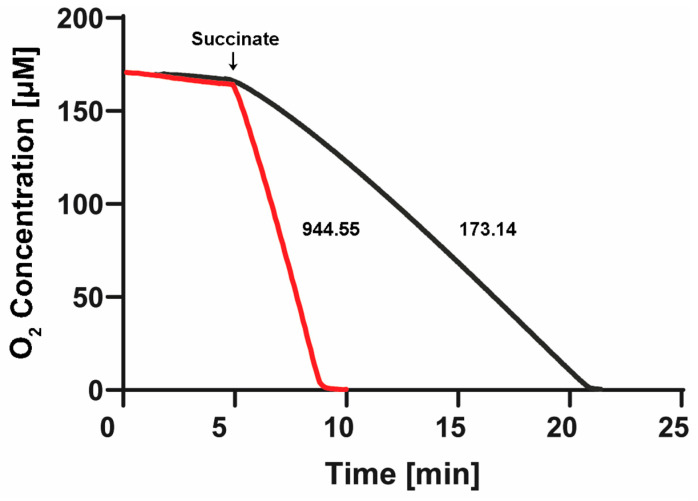
Respiratory rates and oxygen depletion in the Nar-JJ assays of “*aerobic*” and “*anaerobic*” SBP of *P. denitrificans*. One Oroboros 2 mL cell was filled with SBP isolated from *P. denitrificans* cells grown *aerobically* (red trace) and diluted into the same buffer to the same protein concentration used in the *anaerobic* spectrophotometric cells of the Nar-JJ assays, i.e., 0.15 mg/mL (see Section 3). The other Oroboros 2 mL cell was filled identically, but with SBP isolated from *P. denitrificans* cells grown *anaerobically* (black trace). The temperature was kept to 37 °C as in the Nar-JJ assays. After about 5 min of basal respiration without addition of respiratory substrates, 10 mM of succinate was injected, and the rate of oxygen consumption was estimated from the most linear segment of each oxygen decay trace. The red trace was repeated 3 times, obtaining a respiratory rate of 944.5 ± 79 natg O/(min.•mg. pt.), and the black trace was repeated 7 times, obtaining 173.1 ± 2.5 natg O/(min.•mg. pt.). The dissolved oxygen was totally consumed in about 3 min, before the 15 min of oxygen depletion by succinate respiration in the case of “*aerobic*” SBP, and in about 17 min before the 20 min of oxygen depletion used during the Nar-JJ assays with “*anaerobic*” SBP. In the latter experiments, there was no further addition of glucose oxidase + glucose as in the Nar-JJ assays since the oxygen was totally depleted in both Oroboros cells.

After the stabilization of the oxygen concentration in both cells filled with “*aerobic*” SBP or “*anaerobic*” SBP, we confirmed a near-zero basal respiration in the absence of added respiratory substrates (see first 5 min in Figure 6). Afterward, after about 5 min of basal respiration, we added 10 mM of succinate. We carried out three to seven replications of these Oroboros experiments, observing a reproducibly high rate (944.5 ± 79 natg O/(min.•mg. pt.)) of oxygen consumption with SBP isolated from *P. denitrificans* cells grown *aerobically*, (see red trace in Figure 6) but a much slower rate of respiration (173.1 ± 2.5 natg O/(min.•mg. pt.)) with SBP isolated from *P. denitrificans* cells grown *anaerobically* (see black trace of Figure 6). Thus, the SBP isolated from *aerobically* grown *P. denitrificans* cells exhibited a 5.5-fold higher *aerobic* respiratory rate than the SBP isolated from *anaerobically* grown Pd1222 cells. 

More importantly, we clearly confirmed that the residual dissolved oxygen was consumed totally in both SBP preparations before the end of the 15–20 min of the oxygen depletion step. In the SBP isolated from *aerobically* grown *P. denitrificans* cells, the oxygen was consumed in about the first 3 min after succinate addition (see red trace of Figure 6). On the other hand, with the SBP isolated from *P. denitrificans* cells grown *anaerobically*, the oxygen was totally consumed in the first 17 min of succinate reaction. In this case, during the Nar-JJ assays, we observed a higher basal NADH oxidation before NO_3_^−^ addition (see above); thus, we also added a further pre-incubation of 5 min with glucose oxidase + glucose to scavenge the residual oxygen for a total of 20 min of the oxygen depletion step (see Supplementary Figure S3). In conclusion, although we did not inject glucose oxidase + glucose in the Oroboros cell, we can be confident that the residual oxygen was also completely consumed after the further incubation with glucose oxidase during the Nar-JJ assays, i.e., before the 20 min of oxygen depletion after the addition of succinate (see Figure 6 black trace). The high reproducibility, i.e., low standard errors of the respiratory data, allow us to conclude that in the Nar-JJ assays, the dissolved oxygen is fully consumed before the end of the oxygen depletion step with both “*aerobic*” and “*anaerobic*” SBP preparations. These results explain why after the 15 min of the oxygen depletion step, we observed a higher basal NADH consumption exclusively with “*anaerobic*” SBP (see above) but not with “*aerobic*” SBPs. At 15 min of the succinate respiration of “*anaerobic*” SBP, there is still a residual amount of oxygen (Figure 6, black trace) that can be used by the alternate oxidases, which have a higher affinity for O_2_ than Cytochrome-c oxidase or Complex IV, and are overexpressed in *anaerobic* conditions.

However, we resolved this by the further glucose oxidase + glucose oxygen scavenging during the Nar-JJ assays, which completely consumed the near-zero residual oxygen before the 20 min of oxygen depletion step used for “*anaerobic*” SBP (see black trace Figure 6). Taken together, these results confirm that in all the Nar-JJ assays carried out, and after confirming the near zero NADH basal consumption, when NO_3_^−^ is added after the addition of malonate, we determined the Nar activity in essentially total *anaerobic* conditions within the spectrophotometric *anaerobic* reaction cells.

Finally, finding a higher respiratory rate with SBP prepared from *aerobically* grown *P. denitrificans* cells than with SBP obtained *anaerobically* is in concordance with a higher classical respiratory chain activity in *aerobic* conditions, and with a higher alternate denitrifying chain activity in *anaerobic* conditions. Accordingly, the proteomic and transcriptomic analyses had shown a higher expression of the alternate denitrifying chain including Nar, Nir, Nor, and Nos enzymes in *anaerobic* conditions [6,7]. However, the same studies had shown a similar, but not lower, expression of the classical oxygenic respiratory chain, including respiratory complexes I, II, III, IV, and also of complex V or the *P. denitrificans* ATP synthase [6,7]. Thus, the lower oxygenic respiratory chain activity that we observed here (Figure 6) in SBP isolated from *P. denitrificans* cells grown *anaerobically* does not seem to be a result of a lower expression of the respiratory chain complexes I-IV. Therefore, further studies are needed to look more deeply into the precise oxygenic respiratory chain protein expression and/or the control of its activity in *P. denitrificans* grown either in *aerobic* or *anaerobic* conditions. For instance, it is known that Complex IV or Cytochrome-c oxidase is inhibited by nitrite and NO from the denitrifying respiratory chain, and furthermore, this inhibition could transform from a reversible to irreversible inhibition [20]. This could explain, at least in part, how the oxygenic respiratory chain of *P. denitrificans* could be irreversibly inhibited during its *anaerobic* growth, and this inhibition be reflected in the isolated SBP, without a drop in the protein levels of the respiratory chain complexes. However, further studies are needed to ascertain whether this lower oxygenic respiratory chain activity is associated with lower amounts of respiratory complexes expressed, or only to the post-transductional modulation of its electron and/or proton transfer activity rates.

## 3. Materials and Methods

### 3.1. Bacterial Growth

#### 3.1.1. Aerobic Growth

The wild type strain of *Paracoccus denitrificans* Pd1222 was grown *aerobically* in minimal succinate media at 37 °C for 24 h with agitation at 150 rpm. The succinate media was prepared as follows: 50 mM of KH_2_PO_4_, 75 mM of NH_4_Cl, 11.5 mM of Na_2_SO_4_, 1.25 mM of MgCl_2_, 1 mM of citric acid, 10 g/L of succinic acid, and 1 mL/L of stock of concentrated salts. The stock of salts is prepared with the following: 100 mM of CaCl_2_, 90 mM of FeCl_2_, 50 mM of MnCl_2_, 25 mM of ZnCl_2_, 10 mM of CoCl_2_, 5 mM of CuCl_2_, 5 mM of boric acid (H_3_BO_3_), and 10 mM of sodium molybdate (Na_2_MoO_4_). To avoid salt precipitation, it requires adding HCl diluted 1:1 with distilled water to keep the solution transparent. On the other hand, the *aerobic* growth of the wild type strain of *Brucella canis* was carried out overnight for about 15–18 h in similar conditions as those for *P. denitrificans*, but in custom Luria–Bertani (LB) broth, instead of succinate media, with stirring (150 rpm) at 37 °C.

#### 3.1.2. Anaerobic Growth

In the bacterial growth stage, we refer to “*anaerobic”* conditions, but there is no full depletion of oxygen in the bacterial growth media, because there are always residual or trace amounts of dissolved oxygen. We therefore use the terms “*anaerobic*”, “*anaerobically*”, or “*nearly anaerobic*” as synonyms through all this work when referring to the growth media or growth conditions. However, during the Nar-JJ enzymatic assays, we confirmed that the residual dissolved oxygen was fully depleted in the *anaerobic* reaction cells with the Oroboros cell (see Section 2.5).

The *anaerobic* growth of wild type *P. denitrificans* Pd1222 was carried out as follows: a denitrifying culture medium was used, comprising 50 mg/L of succinic acid, 29 mM of Na_2_HPO_4_, 11 mM of NaH_2_PO_4_, 5.4 mM of NH_4_Cl, 0.4 mM of MgSO_4_, 342 μM of EDTA, 15 μM of ZnSO_4_, 51 μM of MgCl_2_, 37.12 mg/L of NaNO_3_, 36 μM of FeSO_4_, 12 μM of Na_2_MoO_4_, 13 μM of CuSO_4_, and 13.5 μM of CoCl_2_. 

An 18 h Luria–Bertani pre-inoculum broth, cultivated at 28°C with agitation at 200 rpm and exhibiting an absorbance of 0.6 (ABS 600 nm), worked as the initial pre-culture. The biomass was subsequently centrifuged (4000 rpm for 10 min) and washed with sterile physiological saline solution (0.9% NaCl). Following this, the biomass was re-suspended in 7000 mL glass bottles, each containing the denitrifying culture medium and 0.6 (ABS 600 nm) of biomass. Subsequently, the bottles were purged with helium for 5 min to remove oxygen, sealed, and then incubated (28 °C, 200 rpm) for 12 h.

#### 3.1.3. Preparation of Membranes and Sub-Bacterial Particles (SBP)

To determine the activity of the membrane-embedded nitrate reductase or Nar enzyme, it was necessary to prepare inside-out inner membranes (sub-bacterial particles (SBP)), since the Nar active site is oriented towards the bacterial cytoplasm (see Figure 1). This was obtained firstly by preparing inner membrane spheroplasts by lysozyme treatment and broken membranes by osmotic shock of the harvested bacteria followed by centrifugation to pellet the SBP [13,21]. This was continued by the sonication of the spheroplasts in ice-cold bath conditions, similar to the preparation of the analogous inside-out submitochondrial particles (SMP) prepared from whole mitochondria [22]. 

#### 3.1.4. Preparation of Plasmatic Membranes from *P. denitrificans*

Firstly, to obtain the spheroplasts, 4–10 L of *P. denitrificans*, grown *aerobically* or *anaerobically* as explained above, were centrifuged at 10,000 rpm for 10 min at 4 °C to harvest the cells. The cell pellets can be weighted to obtain the wet weight of the cells. Afterward, the cell pellets were washed once in 750 mL of buffer A (NaCl 50 mM, Tris 10 mM, pH 7.5) and centrifuged again at 10,000 rpm for 15 min. The harvested and pelleted cells were frozen at −70 °C until they were used to facilitate the cell disruption. Subsequently, the cells were defrosted and re-suspended in 800 mL of buffer B (0.5 M Sucrose, 10 mM Tris-HCl, pH 7.5) + 5 mM EDTA + Lysozyme (264 mg/800 mL of buffer B; the lysozyme pre-dissolved in 30 mL of buffer B + EDTA), and stirred gently for 3 h at 37 °C to break the cell walls. Afterward, the spheroplasts were centrifuged at 10,000 rpm for 15 min at 4 °C. Subsequently, the pellet was re-suspended in buffer C1 (45 mL of 0.55 g Tris/0.25 g ATP +1 tablet of cOmplete™(EDTA-free protease inhibitors cocktail), pH 7.5), homogenized by several cycles of up/down strokes in a potter in a cold ice/water bath, incubated for 20 min at room temperature, and subsequently diluted 1:10 to 450 mL with buffer C2 (5 mM Benzamidine, 1 mM PMSF, +2 tablets of cOmplete™, pH 7.5) to obtain broken membranes by osmotic shock. Afterward, 5 mM of MgCl_2_ and DNAse (≈5 mg/100 g of cells) were added and the mixture was incubated with gentle stirring for 5 min at room temperature. Subsequently, the whole mixture was centrifuged for 1 h at 130,000 rpm at 4 °C. The red pellets (membranes) were re-suspended in the minimal volume of buffer A^+^ and frozen at −80 °C until used. 

#### 3.1.5. Preparation of SBP from Membranes of *P. denitrificans*

These inside-out membrane vesicles or sub-bacterial particles (SBP) were prepared as previously described [13,21]. The membranes of *P. denitrificans*, obtained as explained above, were diluted to 10 mg/mL of protein concentration in a buffer containing 10% glycerol, 250 mM of sucrose, 20 mM of Tris-HCl, 1 mM of EDTA, 0.1 mM of ATP, 2.5 mM of 4-amino benzamidine (PAB), and pH level of 7.5. These membranes were sonicated at 110 Watts in an ice bath for 10 cycles of 10 s, each cycle separated by 50 s of resting time to avoid the warming of the membrane suspension. The sonicated membranes were then centrifuged at 15,000 rpm in the SS-34 rotor (Sorvall) for 20 min at 4 °C. The supernatant was collected and placed on ice, while the pellet was re-suspended in the same buffer to be sonicated again as before. This second sonicated suspension was centrifuged at 15,000 rpm in the SS-34 rotor (Sorvall) for 10 min at 4 °C, and the supernatant was collected. The two supernatants obtained were combined and ultracentrifuged at 45,000 rpm in the 50 Ti rotor for 1 h at 4 °C. The pellets were collected in the smallest possible volume, combined and re-suspended/homogenized by several up/down potter strokes on ice in ≈3 mL of buffer containing 10% glycerol, 250 mM of sucrose, 20 mM of Tris-HCl, 1 mM of EDTA, 0.1 mM of ATP, 2.5 mM of 4-amino benzamidine (PAB), pH level of 7.5, and stored aliquoted in small 25, 50, and 100 μL aliquots at −80 °C to avoid repeated rounds of freezing/thawing of the same SBP aliquots. From one batch of membranes, approximately 150 mg of SBP were obtained.

#### 3.1.6. Determining the Rate of Nitrate Reduction by Coupling Respiratory Complex I to Nar

From the SBP obtained, it was necessary to determine with high accuracy the concentration of protein (in mg/mL) present in the final suspension obtained; this is to determine the specific activity (μmol/(min.•mg.pt.)), i.e., μmol of NO_3_^−^ reduced per milligram of the total protein present in SBP per minute. This can be completed by the linear regression of any high accuracy protein determination method using the appropriate protein titration curve. We suggest the Lowry method [23] and its trichloroacetic acid (TCA) precipitation modification [24], which removes any putatively interfering substance from the SBP present in the samples.

The specific nitrate reductase (Nar) activity was measured by the increased oxidation of NADH to NAD^+^ from Complex I at 340 nm in *anaerobic* cells induced by NO_3_^−^ and observed as an accelerated negative slope. To achieve *anaerobic* conditions, 300 µg of SBP in “Nar reconstitution buffer” (250 mM Sucrose, 100 mM KCl, 20 mM MES-TRIS, pH 7.5) were pre-incubated with 10 mM of succinate at 37 °C, to consume the dissolved oxygen by succinate oxidation through the aerobic respiratory chain. Afterward, to block further succinate oxidation by the respiratory Complex II, 10–20 mM of malonate, an inhibitor of CII, was added to the reaction medium. When the basal NADH oxidation before addition of NO_3_ was still too high after the 15 min of succinte respiration step (as in the case of SBP isolated from *anaerobilcally* grown *P. denitrificans* cells) a further 5 minutes incubation in the presence of glucose oxidase + glucose removed essentially all traces of oxygen and leaved a nearly zero NADH oxidation slope (see Supplementary Figure S3 and Figure 3). Full oxygen depletion in these succinate respiration steps in the 2 mL *anaerobic* cells were confirmed by Oroboros oximetry (see Section 2.5 and Figure 6). At this stage, by adding NO_3_^−^ to the reaction cells under essentially full *anaerobic* conditions, the only available electron acceptor was nitrate, the natural substrate for the enzyme nitrate reductase or Nar. For this determination, an optimal saturating nitrate concentration of 5 mM was obtained. The NO_3_^−^-induced increase in the linear slope of the NADH absorbance decay was taken as the rate of the NO_3_^−^ reduction carried out by Nar. The first nearly horizontal slope after the NADH addition and before the addition of NO_3_^−^ was taken as the basal NADH oxidation, and this slope was subtracted from the faster NADH decay slope obtained after the NO_3_^−^ addition to obtain the specific rate of Nar activity.

## 4. Conclusions

*Issue*: The description of a relatively simple novel coupled CI/Nar assay to determine in real-time the specific activity of the membranous nitrate reductase or Nar is here thoroughly described for the first time and for its general application in laboratories interested in measuring the endogenous Nar activity of denitrifying bacteria. *Key findings*: The advantages of this method are several: it is very specific and reliable for the determination of the endogenous Nar activity in SBP, and it works with endogenous natural substrates, not artificial ones, and with the endogenous respiratory Complex I as the only coupling enzyme. *Perspectives*: The method can be applied to *P. denitrificans* and other pathogenic denitrifying bacteria such as *Brucella canis*, and can be assayed for *Salmonella enterica*, pathogenic *E. coli* strains, etc. This will be useful in the future in the search for novel antimicrobials targeting Nar, and in other biotechnological and water-treatment applications by bacterial denitrification.

## 5. Important Practical Points

To our knowledge, a similar method has not been previously described in such detail, other than a similar approach that was briefly mentioned in a previous publication but without a formal validation and description [12]. This method does not require additional coupling enzymes, so this makes it relatively low-cost and easy to apply. The total reaction time is about 25–30 min, 15–20 min of de-oxygenation with succinate respiration, plus about 10 min of total reaction including the basal step and the nitrate-reduction by Nar coupled to the NADH decay. So, it is relatively fast once the SBP and the proper reaction media and concentrated stocks are prepared. The most important step is the preparation of spheroplasts, the membranes by osmotic shock, and the further preparation of the inside-out vesicles or SBP. Taking good care during these steps will ensure the better kinetic and thermodynamic coupling of Complex I with the Nar enzyme. So, it is suggested to ensure that the detailed steps in the preparation of membranes and SBP are followed carefully, otherwise membranes could be unstable and thus the enzymes could inactivate quickly. The preparation of SBP in ice-cold baths and cold (4 °C) centrifugations are essential to prevent the heating or warming of the membranes and SBP during their preparation processes. It is also essential to ensure the correct preparation of buffers is completed, along with protease inhibitors where indicated. SBP should be aliquoted just after their preparation and stored at −80 °C to defrost single aliquots every day or when every Nar-JJ assay is to be carried out, to avoid several rounds of freezing and thawing of the same SBP tubes. It is also important to inject the smallest aliquots as possible (1–20 μL) into the already *anaerobic* cells containing substrates, or inhibitors, to prevent the injection of dissolved oxygen into the *anaerobic* cells as much as possible. Taking care of this decreases the initial basal NADH decay without NO_3_^−^ to a near-zero slope very quickly. This near-zero basal NADH consumption before the NO_3_^−^ addition should be always confirmed and subtracted from the faster Nar-associated NADH decay coupled to the nitrate reduction. If the basal NADH consumption is still too high without NO_3_^−^, the addition of glucose oxidase + glucose after the oxygen exhaustion with succinate and the addition of malonate, as described in the Section 3, quickly slows down the basal NADH decay to near zero to be able to start the Nar reaction. The KNO_3_ salt should be used for the preparation of substrate stocks for the Nar-JJ assay, as it works much better than the NaNO_3_ salt, but the latter works well for the preparation of *anaerobic* growth media with nitrate, as described in the Section 3. As mentioned, this method has been described here in detail for SBP isolated from *P. denitrificans* and *Brucella canis*, both α-proteobacteria, but it should be suitable to be applied to other non-α-proteobacteria denitrifiers and enteric pathogens such as *Salmonella enterica* or pathogenic *Escherichia coli*, among others, as long as the NADH consumption will be carried out exclusively by Complex I, or stoichiometrically coupled 1:1 to the Nar reduction of nitrate. This method could probably also be adapted to other bacteria lacking Complex I, and having Complex II for the oxygen exhaustion step, such as *Vibrio cholerae* among others, provided that the proper coupling of alternate NADH and/or NQR oxidases with the Nar enzyme will be confirmed. If the bacterium of interest lacks Complex II, the anaerobic conditions could be obtained as mentioned above, by the N_2_ current inside the *anaerobic* cells. In all cases, it should preferably and firstly be validated that the Complex I, or alternate NADH oxidases, are not the rate-limiting steps, but the Nar reaction should be the slowest one to calculate reliable Nar activities.

## 6. Patents

This work is associated to the accepted patent with registration number: MX/a/2017/016448.

## Data Availability

Data is contained within the article and Supplementary Materials.

## Supplementary Materials

The following supporting information can be downloaded at: https://www.mdpi.com/article/10.3390/ijms25189770/s1.
