# Supplementary material for: A New Real-Time Simple Method to Measure the Endogenous Nitrate Reductase Activity (Nar) in Paracoccus denitrificans and Other Denitrifying Bacteria
Source: Int J Mol Sci. 2024 Sep 10;25(18):9770. doi: 10.3390/ijms25189770 (PMC11431489; doi:10.3390/ijms25189770)
Supplement: Supplementary file 1 [file ijms-25-09770-s001.zip › ijms-3141931-supplementary.pdf]

Supplementary Material

García-Trejo, J.J. et al. "A new real-time simple method to measure the endogenous nitrate reductase activity (Nar) in *Paracoccus denitrificans* and other denitrifying bacteria".

Supplementary Figure S1

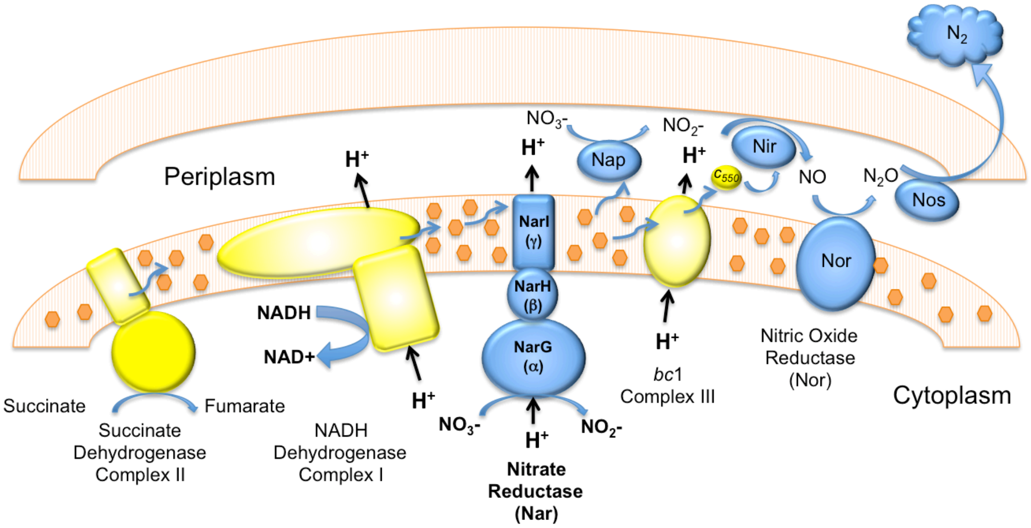

Supplementary Figure S1. The anaerobic denitrification respiratory chain of *Paracoccus denitrificans*. The 5 denitrification dissimilatory enzymes of *P. denitrificans* are shown in blue. The classical aerobic respiratory complexes (CI, CII, and CII) are shown in yellow. The quinone pool is shown as orange hexagons inside the inner plasma membrane of the bacterium. The electron transfer reactions are shown by blue arrows. The denitrication alternate respiratory chain of *P. denitrificans* starts with the Nitrate Reductase or Nar at the center of the drawing. However, the redox loop connected to Nar *in vivo* starts with electrons derived from NADH or succinate transferred to the quinone pool through the classical aerobic respiratory chain with Complex I or Complex II, respectively. These two complexes, CI and CII, are not shown according to their redox potential, rather they are accommodated to show the direct coupling of Complex I and Nar, that is essential for the Nar-JJ method described here. This coupling occurs through the quinone pool since the quinol which is reduced by CI or CII is oxidized by Nar. *P. denitrificans* can also reduce the periplasmic NO_3^- to NO_2^- with the water-soluble Nitrate Reductase or Nap. On the other hand, and as shown by the black arrows, CI, CIII, and Nap pump protons to the periplasmic space coupled to their oxidation-reduction reactions to build up the electrochemical proton gradient ($\Delta\mu_{H+}$) across the inner bacterial membrane. Proton pumping is not shown with its specific stoichiometry for each redox pump, but only indicated. Both, NO_3^- and NO_2^- can cross the inner membrane through specific transporters (not shown for simplicity). NO_2^- is further reduced to Nitric Oxide (NO) by the periplasmic Nitrite Reductase (Nir), and the latter is reduced to nitrous oxide (N_2O) by the membranal nitric oxide reductase (Nor). Subsequently, the nitrous oxide (N_2O) is transformed to molecular dinitrogen (N_2) by the periplasmic nitrous oxide reductase (Nos). Cytochrome *c*550 (or pseudoazurin (Paz), not shown) is believed to reduce Nir, Nor, and Nos for these final electron transfer steps.

The membranous *Escherichia coli* nitrate reductase or Nar has been structurally resolved by X-ray crystallography [9]. The Nar enzyme of *E. coli* is homologous to that of *P. denitrificans*, although the latter α-protobacterium has the full denitrifying respiratory chain from nitrate reduction to dinitrogen production (see Supplementary Figure 1), whereas *E. coli* (and other non-fully denitrifying bacteria) only possesses the Nar enzyme. Nevertheless, both Nar enzymes from *P. denitrficans* and *E. coli* are ortologous and thus share the main core structure of three subunits. The crystal structure of the Nar enzyme from *E. coli* (PDB_id 1Q16) can be seen in Supplementary Figure 2A, with the α subunit (or NarG) holding the functional prosthetic groups for nitrate reduction, i.e. the Molibdo bis-(Molibdopterin Guanine Dinucleotide) or Mo-bisMGD, and an iron-sulfur cluster [4Fe-4S]. The β subunit or NarH, connects structurally and functionally the α (NarG) subunit with the transmembrane γ subunit, or NarI. The β subunit or NarH contains one [3Fe-4S] and three [4Fe-4S] iron-sulfur centers, arranged in the structure according to their redox potential, which allows the transfer of electrons through the protein (Supplementary Figure 2A). The NarI or γ subunit is the transmembranal protein holding the two heme-*b* groups that transfer electrons from the membrane quinone pool (Supplementary Figure 2A). In the sense of nitrate reduction, the connection carried out by the β subunit is achieved by its iron-sulfur clusters that carry the electrons from the *b* hemes of the γ subunit to the prosthetic groups of the α subunit, which deliver the electrons to NO_3^- (from bottom to top in Supplementary Figures 2 A and C). Although the proton pumping mechanism of this Nar enzyme is still unresolved in detail, it is known that the enzyme couples the oxidation of reduced quinol to the reduction of nitrate through a redox loop mechanism [8] involving the two *b*-type heme groups in its transmembrane γ subunit. A structural model of the Nar enzyme from *P. denitrificans* was constructed from the available models from the AlphaFold server, and assembled in PyMol, with further refinement in Chimera. The *P. denitrificans* Nar model or PdNar (Supplementary Figure 1C) superimposes very well (Supplementary Figure 2B) with the original *E. coli* Nar structure or EcNar (PDB_ID 1Q16, Supplementary Figure 2A).

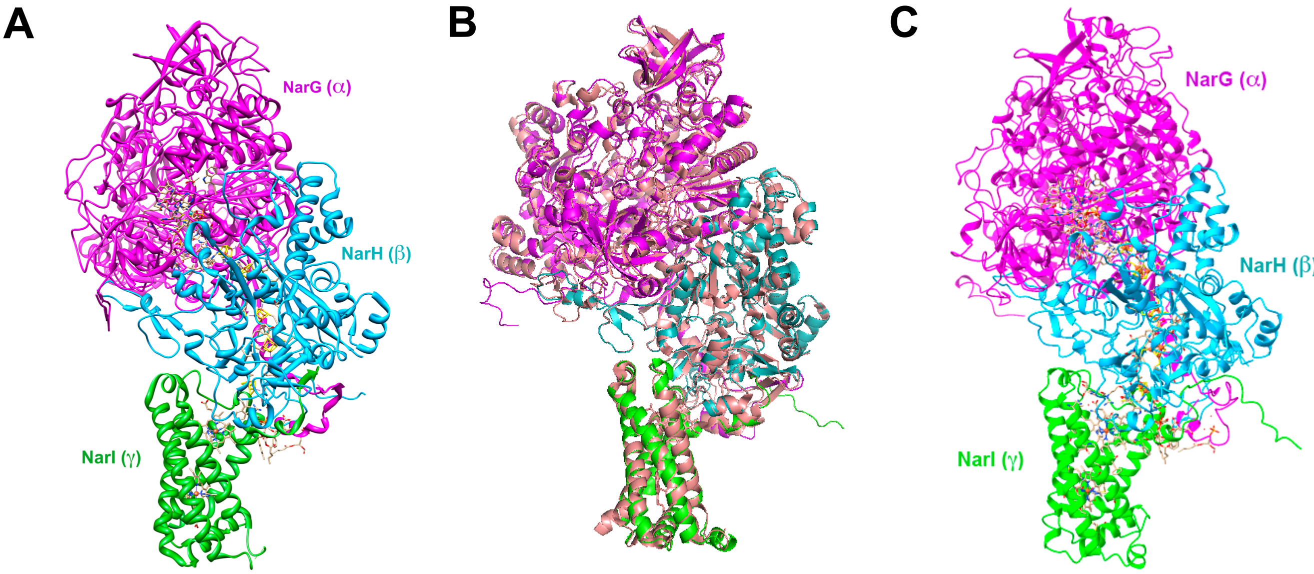

Supplementary Figure S2. Structures of the Nar enzymes from *E. coli* and *P. denitrificans*. A) Crystallographic structure of the Nar enzyme from *E. coli* (EcNar) (PDB_id 1Q16) shown in ribbons. Colour codes: Magenta NarG (α); Blue NarH (β); Green NarI (γ). Redox groups MGD (NarG or α), FeS clusters (NarH or β) and heme groups (NarI or γ) are shown in yellow sticks. The path of electrons from oxidation of QH_2 in the membrane at NarI or γ (green) up to NarH or β FeS clusters (blue) and finally to the "top" at MGD of Nar G or α can be traced as an internal right-curved yellow electron transfer path of prosthetic group. B) Superposition of the *E. coli* Nar enzyme structure (PDB_id 1Q16) in salmon colour, with the model of the structure of the *P. denitrificans* Nar enzyme (PdNar) constructed and assembled from the available AlphaFold structures of the Nar enzyme from *P. denitrificans* (PdNar), colour code of PdNar same as in A), both structures are nearly identical and superimpose very well. C) Modelled structure of the PdNar enzyme, with the same colour code as in A) and B). The same right-curved electron path can be traced in yellow redox prosthetic groups as in A).

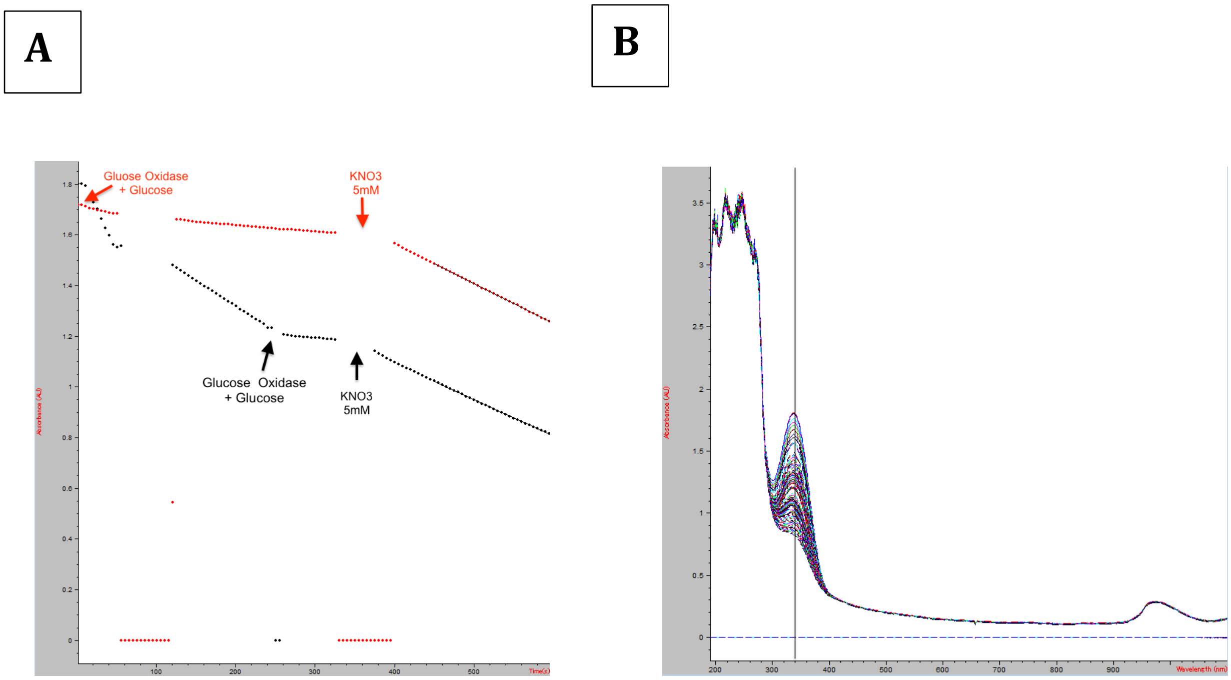

Supplementary Figure S3. Residual oxygen depletion by glucose oxidase + glucose during Nar-JJ assays and NADH decay spectra. Original spectrophotometric traces (A) and spectra (B) are shown. A) Two anaerobic spectrophotometer cells were preincubated by 15 minutes with SBP isolated from *anaerobically* grown *P. denitrificans* Pd1222 cells with 10 mM succinate to consume the dissolved oxygen, and after the completion of the 15 minutes of succinate respiration, 20 mM malotate was added to stop succinate respiration. In the red trace, we added 5 µL of a concentrated (20 mg/ml) glucose oxidase stock to the anaerobic cells containing 10 mM glucose at time zero. Here the NADH decay was slowed down to a minimal basal slope within the first 5 minutes (aprox. 300 seconds) after adding glucose oxidase +glucose. The further addition of 5mM KNO_3 started the CI-Nar coupled reaction. The black lines show the linear regression fitting to calculate the slope of the NADH decay of the Nar reaction. The Nar specific activity is calculated after subtraction of the nearly zero slope after addition of glucose oxidase + glucose. In the black trace glucose oxidase + glucose were added, not from the beginning at time zero, but after about 250 seconds of a faster basal NADH decay produced by the residual oxygen, and presumably the highly expressed alternate oxidases with higher oxygen affinity than Complex IV. The addition of glucose oxidase + glucose scavenged the residual oxygen, and thus slowed down immediately the basal NADH decay, and the further addition of 5 mM nitrate started the coupled CI-Nar reaction. B) The absorbance spectra of these two samples as a function of time. The vertical line at 340 nm shows the NADH absorbance peak, the spectra shows de decay of the NADH peak at 340 nm as a function of time as it is transformed to NAD^+.

References

The cited references are enlisted in the main text of this paper.
